# Hepatic methotrexate-associated lymphoproliferative disorders identified by multiple liver tumors: a case report and review of the literature

**DOI:** 10.1186/s13256-019-2135-3

**Published:** 2019-06-27

**Authors:** Ryohei Ono, Tomohiro Kumagae, Haruki Uojima, Shinichi Teshima, Madoka Kudo, Izumi Kitagawa, Masaki Yoshizawa

**Affiliations:** 1 Department of General Internal Medicine, Shonan Kamakura General Hospital, 1370-1 Okamoto, Kamakura city, Kanagawa 247-8533 Japan; 2 Department of Gastroenterology, Internal Medicine, Kitasato University School of Medicine, 1-15-1 Kitasato, Minami-ku, Sagamihara, Kanagawa 252-0375 Japan; 3 Department of Diagnostic Pathology, Shonan Kamakura General Hospital, 1370-1 Okamoto, Kamakura city, Kanagawa 247-8533 Japan; 4 Department of Rheumatology, Shonan Kamakura General Hospital, 1370-1 Okamoto, Kamakura city, Kanagawa 247-8533 Japan

**Keywords:** MTX-LPD, Methotrexate-associated lymphoproliferative disorders, Fever, Rheumatoid arthritis, Liver tumor

## Abstract

**Background:**

Methotrexate, an immunosuppressant, is widely used as the standard therapeutic drug for rheumatoid arthritis. With the increasing frequency of use of methotrexate, adverse effects of methotrexate have been reported, one of which is known as methotrexate-associated lymphoproliferative disorders. The etiology of hepatic methotrexate-associated lymphoproliferative disorders remains largely unknown. To date, there have only been ten cases of hepatic methotrexate-associated lymphoproliferative disorders reported in the English literature and a case report is very rare.

**Case presentation:**

An 82-year-old Japanese man with rheumatoid arthritis treated with methotrexate presented with fever. Contrast-enhanced computed tomography showed multiple hypovascular nodules in his liver, spleen, and lung, and para-aortic lesions. Endoscopic ultrasound-guided fine-needle aspiration biopsy for liver tumors was performed, and pathological results identified cluster of differentiation 20-positive lymphocytes. Discontinuance of methotrexate led to regression of the nodules and a final definitive diagnosis of methotrexate-associated lymphoproliferative disorders was made.

**Conclusions:**

We review 11 reported cases of hepatic methotrexate-associated lymphoproliferative disorders including the present case. Physicians should discontinue methotrexate in patients with rheumatoid arthritis treated with methotrexate when elevated soluble interleukin-2 receptor and hypovascular lesions in contrast-enhanced computed tomography are confirmed considering the possibility of methotrexate-associated lymphoproliferative disorders.

## Background

Methotrexate (MTX), an immunosuppressant, is widely used as the standard therapeutic drug for rheumatoid arthritis (RA) [1]. With the increasing frequency of use of MTX, adverse effects of MTX have been reported, one of which is known as MTX-associated lymphoproliferative disorders (MTX-LPD) [2]. The etiology of hepatic MTX-LPDs remains largely unknown. We describe a rare case of hepatic MTX-LPD in a patient with RA, in which multiple hepatic lesions developed during the treatment with MTX and there was regression of the lesions after its discontinuation. We reviewed the literature on MTX-LPD in the liver and summarized the relevant characteristics.

## Case presentation

An 82-year-old Japanese man with a history of RA presented with fever and malaise. His medical history revealed RA for 10 years. He had been undergoing treatment with MTX, prednisolone, and bucillamine for 9 years and 6 months (MTX 12 mg/week, prednisolone 2.5 mg/day, and bucillamine 100 mg/day). The MTX dose was initially 4 mg/week; however, the RA symptoms were not well controlled. Therefore, the dose was gradually increased to MTX 12 mg/week, and the total dosage was 3454 mg. Other medical history included benign prostatic hyperplasia and gout. He smoked cigarettes at 1.5 packs per day for 20 years but did not drink alcohol. His family history was unremarkable.

**Fig. 1:**
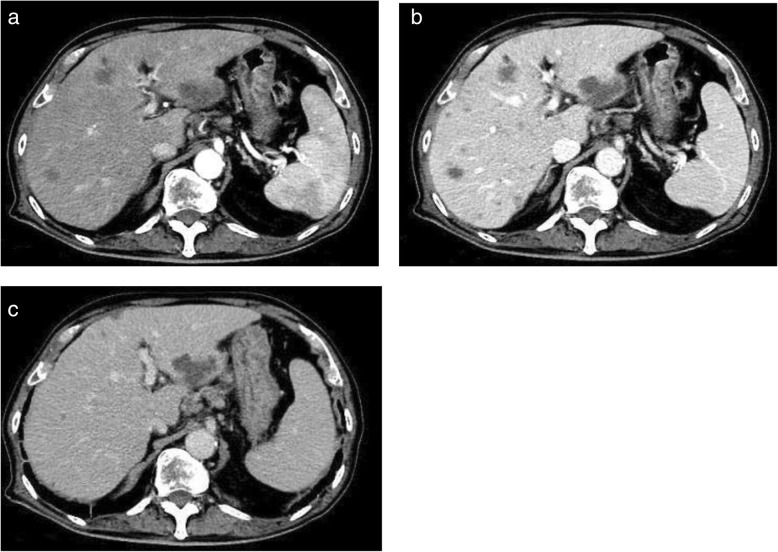
Contrast-enhanced computed tomography image of the abdomen showing multiple hypovascular nodules in the liver, spleen, and para-aortic lesions. **a** Arterial phase, **b** portal vein phase, **c** late phase

On presentation, he was alert, and his Glasgow Coma Score was 15. His body mass index was 23.7 kg/m^2^ with no noticeable body weight changes. He had a fever, but his other vital signs were stable: blood pressure, 128/57 mmHg; pulse, 88/minute; body temperature, 39.2 °C; respiratory rate, 18/minute; and oxygen saturation, 98%. No particular abnormal physical findings were noted other than chronic swelling of his wrists and ulnar deviation of his digits, although he was adequately treated. Furthermore, no enlargement of superficial lymph nodes was observed. Laboratory studies revealed findings of elevation of C-reactive protein (CRP) and soluble interleukin-2 receptor (sIL-2R). Serum hepatobiliary enzymes, rheumatoid factor, and anti-cyclic citrullinated peptide antibody were also elevated (Table 1). Three months prior to the admission, his serum hepatobiliary enzymes were within normal range: aspartate aminotransferase, 20 IU/L; alanine aminotransferase, 18 IU/L; lactate dehydrogenase (LDH), 180 IU/L; γ-glutamyl transpeptidase, 26 IU/L; and alkaline phosphatase, 323 IU/L. No lymphoma cells were detected in his peripheral blood. Tests for viral markers revealed: hepatitis B surface antigen negative, hepatitis C antibody negative, and Epstein–Barr virus (EBV) viral capsid antigen antibody immunoglobulin G and EBV anti-Epstein–Barr nuclear antigen titers were elevated (1:320 and 1:20, respectively). Serum carbohydrate antigen 19-9, sialyl Lewis X-I antigen, and neuron-specific enolase were slightly elevated. Abdominal ultrasonography showed several hypoechoic masses in his liver. We performed contrast-enhanced computed tomography (CT) of his chest and abdomen to identify the cause of his fever, which revealed multiple nodular masses without enhancement in his lung, liver, spleen, and para-aortic lesions (Fig. 1). He was admitted for further evaluation to make a definitive diagnosis.

**Table 1:** Summary of the laboratory data on admission

Complete blood count			
White blood cells	44 × 10^2^/*μL* (30–97 × 10^2^/*μL*)	Ferritin	636.1 ng/mL (13–277 ng/mL)
Neutrophils	75.1% (36.6–79.9%)	Glucose	111 mg/dL (70–110 mg/dL)
Hemoglobin	11.1 g/dL (13.1–17.6 g/dL)	HbA1c	5.8% (4.6–6.2%)
Hematocrit	32.8% (38.1–50.8%)	C-reactive protein	6.66 mg/dL (0–0.5 mg/dL)
Mean cell volume platelets	84.1 fL (84.6–100.6fL)	sIL-2R	4090 U/mL (127–582 U/mL)
Platelet counts	21.1 × 10^4^/*μL* (12.4–30.5 × 10^4^/*μL*)	Rheumatoid factor	490.76 U/mL (< 15 U/mL)
Coagulation		MMP-3	49.4 ng/mL (36.9–121.0 ng/mL)
PT-INR	1.14 (0.89–1.12)	Anti-CCP antibody	118.1 U/mL (0–4.4 U/mL)
APTT	29.5 seconds (23.6–31.3 seconds)	HBs Ag	(−)
Biochemistry		HCV-Ab	(−)
Total bilirubin	0.5 mg/dL (0.1–1.2 mg/dL)	Tumor marker	
Aspartate aminotransferase	89 IU/L (12–35 IU/L)	AFP	1.3 ng/mL (0–10 ng/mL)
Alanine aminotransferase	76 IU/L (6–40 IU/L)	PIVKA-II	13.49 mAU/mL (0–39 mAU/mL)
Lactate dehydrogenase	346 IU/L (119–229 IU/L)	CA19-9	130.9 U/mL (0–37 U/mL)
γ-glutamyl transpeptidase	82 IU/L (0–48 IU/L)	SCC antigen	0.6 ng/dL (< 2.0 ng/mL)
Alkaline phosphatase	608 IU/L (115–359 IU/L)	CEA	2.6 ng/mL (0–6 ng/mL)
Blood-urea-nitrogen	23.6 mg/dL (7.4–19.5 mg/dL)	SLX	54.7 U/mL (0–38 U/mL)
Creatinine	0.87 mg/dL (0.5–1.2 mg/dL)	NSE	16.6 ng/mL (0–12 ng/mL)
Total protein	6.5 g/dL (6.4–8.3 g/dL)	CYFRA	2 ng/mL (0–2 ng/mL)
Albumin	2.5 g/dL (3.8–5.2 g/dL)	Virus titer	
Na	135 mEq/L (135–147 mEq/L)	EBV-VCA IgG	320fold
K	4.2 mEq/L (3.4–4.8 mEq/L)	EBV-VCA IgM	<tenfold
Cl	103 mEq/L (98–110 mEq/L)	EBV-VCA IgA	<tenfold
Fe	30 μg/mL (60–210 μg/mL)	EBV-EBNA	20fold

*AFP* alpha-fetoprotein, *anti-CCP* antibody anti-cyclic citrullinated peptide antibody, *APTT* activated partial thromboplastin time, *CA19-9* carbohydrate antigen 19-9, *CEA* carcinoembryonic antigen, *CYFRA* cytokeratin fragment, *EBV-EBN*A Epstein–Barr virus-Epstein–Barr virus nuclear antigen, *EBV-VCA* Epstein–Barr virus-viral capsid antigen, *HbA1c* glycated hemoglobin, *HBs Ag* hepatitis B surface antigen, *HCV-Ab* hepatitis C virus antibody, *IgA* immunoglobulin A, *IgG* immunoglobulin G, *IgM* immunoglobulin M, *MMP-3* matrix metalloproteinase 3, *NSE* neuron-specific enolase, *PIVKA-II* protein induced by vitamin K absence-II, *PT-INR* prothrombin time-international normalized ratio, *SCC* squamous cell carcinoma, *sIL-2R* soluble interleukin-2 receptor, *SLX* sialyl Lewis X-I antigen

**Fig. 2:**
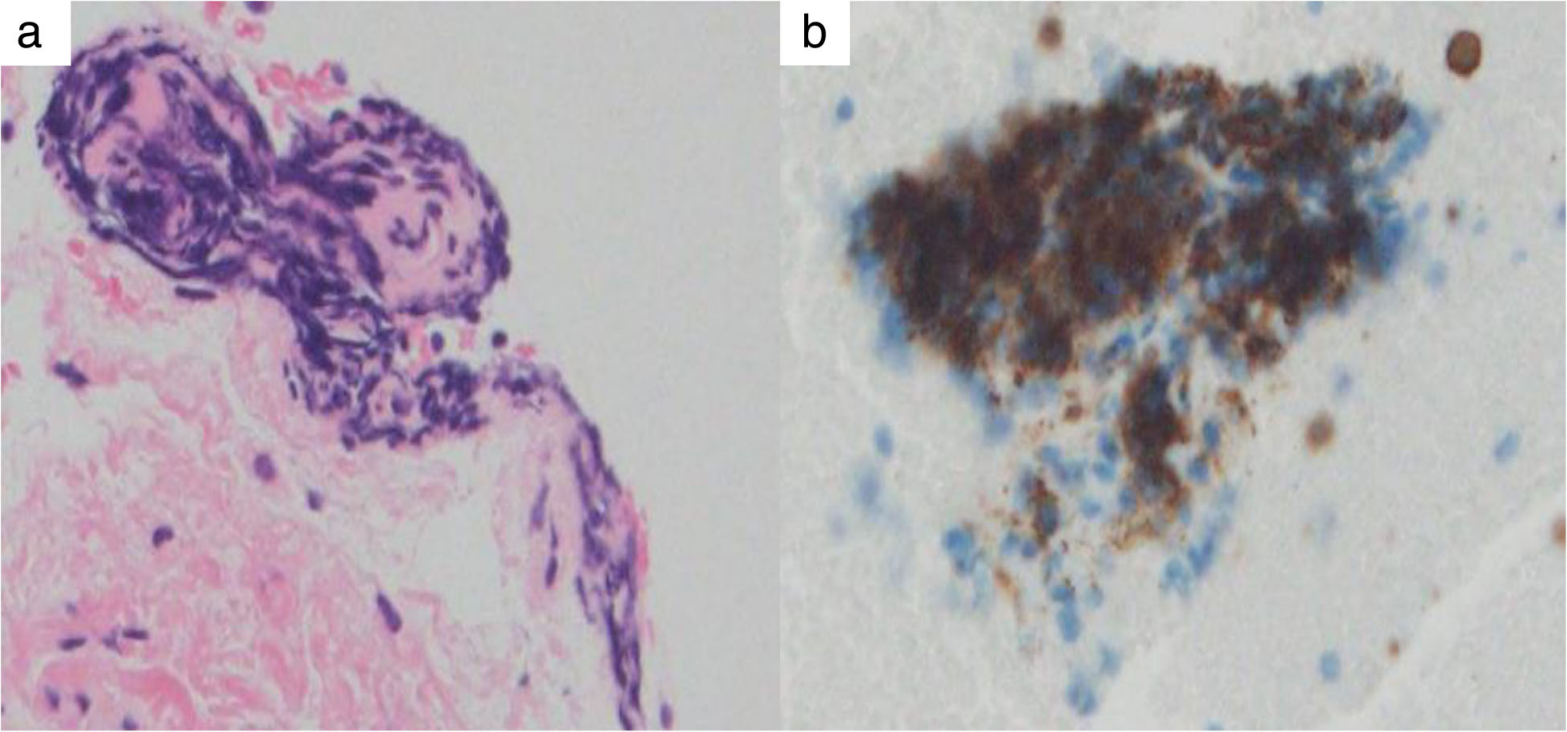
Pathological results obtained from endoscopic ultrasound-guided fine-needle aspiration biopsy for liver tumors indicated B cell lymphoma. **a** Hematoxylin and eosin stain × 200. **b** Cluster of differentiation 20 immunostain × 200

**Fig. 3:**
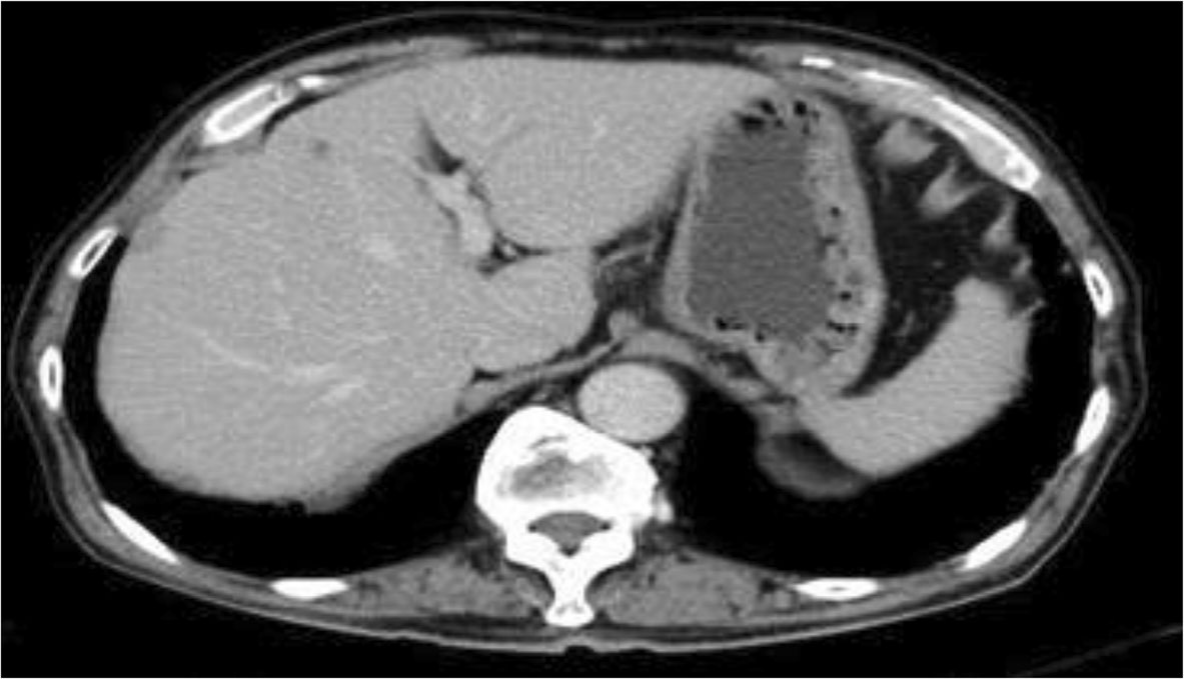
Contrast-enhanced computed tomography image of the abdomen 3 months after the initial visit showing regression of tumors

Soon after admission, MTX was discontinued considering the possibility of MTX-LPD. The differential diagnoses included hepatic malignant lymphoma, hepatocellular carcinoma, intrahepatic cholangiocarcinoma, and metastatic liver cancers because sIL-2R and some tumor markers were elevated. Upper and lower gastrointestinal endoscopy were performed, but no malignant findings were noted. Although percutaneous liver biopsy was performed, it failed to approach the mass. Subsequently, endoscopic ultrasound-guided fine-needle aspiration biopsy for liver tumors was performed. Histological examinations of the specimens revealed cluster of differentiation 20 (CD 20)-positive B cell lymphocytes with necrotic tissue and no malignant findings (Fig. 2). The EBV-encoded small ribonucleic acid by *in situ* hybridization of liver specimen was negative. The tumor began shrinking after MTX was discontinued, and CT of his lung and abdomen 3 months later revealed the disappearance of most of the tumors spontaneously and dramatically (Fig. 3). Serum levels of sIL-2R, LDH, and carbohydrate antigen 19-9 also improved spontaneously (314 U/mL, 120 IU/L, and 74.3 U/mL, respectively), and serum levels of sialyl Lewis X-I antigen and neuron-specific enolase were normalized as well. Serum level of liver enzymes also became within normal range in our case. A definitive diagnosis of MTX-LPD was made based on the clinical course and pathological findings. After withdrawal of MTX, he started abatacept for his RA and experienced no deterioration in his joints. There has been no sign of recurrence for 6 months.

## Discussion

LPD is widely known to be associated with MTX use in patients with RA and EBV activation [3]. LPD developed in patients with autoimmune disease receiving immunosuppressive therapy such as MTX are categorized as other iatrogenic immunodeficiency-associated LPD. According to the World Health Organization classification of tumors of hematopoietic and lymphoid tissue, other iatrogenic immunodeficiency-associated LPD are lymphoid proliferations or lymphomas that arise in patients treated with immunosuppressive drugs for autoimmune diseases or conditions other than in post-transplant settings [4]. Primary hepatic lymphoma is very rare and accounts for 0.4% of extranodal non-Hodgkin’s lymphoma and 0.016 to 0.06% of all non-Hodgkin’s lymphoma [1, 4]. To date, there have only been ten cases of hepatic MTX-LPD reported in the English literature by PubMed [1–3, 5–11]. We reviewed all 11 cases including the present case (Table 2).

**Table 2:** Reported cases of patients with methotrexate-associated lymphoproliferative disorders in the liver

Case	Ref.	Year	Age	Sex	Duration of MTX (months)	Total MTX dose (mg)	Pathology	Treatment	Outcome
1	5	2006	48	F	39	1268	DLBCL	Discontinuation of MTX	Remission
2	2	2013	69	M	84	Not described	Lymphocytes and interstitial fibrosis	Discontinuation of MTX	Remission
3	7	2014	67	F	> 72	1872	DLBCL	Discontinuation of MTX R-THP-COP 6 courses	Remission
4	1	2015	64	M	24	8–14 mg/week for 2 years	DLBCL	Discontinuation of MTX R-CHOP 8 courses	Remission
5	8	2015	56	F	84	5304	DLBCL	Discontinuation of MTX R-CHOP 6 courses	Remission
6	3	2015	70	M	96	5004	B cell lymphoma	Hepatectomy Discontinuation of MTX	No recurrence
7	9	2015	68	F	96	Not described	Hodgkin’s lymphoma	Discontinuation of MTX	Died
8	6	2016	63	M	120	3110	DLBCL	Discontinuation of MTX	Remission
9	10	2017	65	F	84	Not described	DLBCL	Hepatectomy Discontinuation of MTX	No recurrence
10	11	2017	88	F	72	1932	Hodgkin’s lymphoma	Discontinuation of MTX Chemotherapy	Recurrence but remission
11	–	2018	82	M	114	3454	B cell lymphoma	Discontinuation of MTX	Remission

*DLBCL* diffuse large B cell lymphoma, *F* female, *M* male, *MTX* methotrexate, *R-CHOP* rituximab, adriamycin, cyclophosphamide, vincristine, and prednisolone, *Ref.* references, *R-THP-COP* rituximab, cyclophosphamide, pirarubicin, vincristine/prednisolone

Table 2 lists the clinical features of MTX-LPD. The characteristics of these cases showed the median age at presentation was 67 years of age (range, 48 to 88), and the female-to-male ratio was 1.2 (female, 6; male, 5). All patients had a history of RA treated with MTX, and the median age when diagnosed as having RA was 53 years (range, 29 to 74). The majority of patients had long-standing RA, although the duration of RA of some cases was within 3 years (median duration, 10 years; range, 2 to 38 years). The median interval between starting MTX and the development of LPD was 84 months (range, 24 to 120 months). The median total dose of MTX was 3110 mg (range, 1268 to 5304 mg). Other remarkable predisposing factors were tuberculosis, chronic kidney disease, and hepatitis B. All those diseases could cause immunosuppressive conditions. At the time of diagnosis of MTX-LPD, five patients (45.5%) presented with organ involvement other than liver, including lung (*n* = 2), spleen (*n* = 2), abdominal para-aortic lymph node (*n* = 2), adrenal grand (*n* = 2), cervical lymph node (*n* = 1), and retroperitoneal lymph node (*n* = 1). Six cases (54.5%) only had liver involvement. To date, most of the reported cases of MTX-LPD in the liver, including the present case, have had multiple lesions. Among the 11 reported cases, eight had multiple lesions compared to three cases of single lesions.

The laboratory findings are limited because some reports do not mention them. Considering as many data as we could obtain, liver function enzymes were slightly elevated, while sIL-2R, LDH, and CRP were relatively increased. The median values at the presentation were 1.9 mg/dL of total bilirubin (range, 0.5 to 2.9 mg/dL), 90.5 IU/L of aspartate aminotransferase (range, 34 to 153 IU/L), 71.5 IU/L of alanine aminotransferase (range, 23 to 92 IU/L), 4067/mL of sIL-2R (range, 1062 to 8850 U/mL), 557 IU/L of LDH (range, 144 to 2963 IU/L), and 7.555 mg/dL of CRP (range, 6.66 to 28.65 mg/dL). In particular, all cases were associated with a high value of more than 1000 U/mL for sIL-2R. As for the possible mechanisms of elevated liver enzymes, there are three major causes. The first one is tumor involvement of the small intrahepatic biliary tree, which can cause progressive cholangitis, duct necrosis, and liver function impairment. The second mechanism is that extensive infiltration of sinusoids and hepatic vasculature by the tumor causes diffuse hepatic necrosis. Finally, cytokines such as interleukin 2, which are particularly secreted from tumor cells, might damage interlobular bile ducts and cause portal fibrosis either directly or by an immunological mechanism [12].

As previously stated, about half of the cases involved only the liver, so the imaging modality is important for the diagnosis. Some previous reports suggested imaging studies, especially ultrasonography and CT, are helpful for diagnosis. However, other types of imaging modalities, such as magnetic resonance imaging, fluorodeoxyglucose-positron emission tomography (FDG-PET), and gallium scintigraphy have been reported. CT was used in almost all cases (*n* = 11), followed by ultrasound (*n* = 8), FDG-PET (*n* = 4), magnetic resonance imaging (*n* = 3), and gallium scintigraphy (*n* = 1). For the patient in the present case report, contrast-enhanced CT scans featured multiple hypovascular lesions, but it is difficult to distinguish hepatic lymphoma from hepatocarcinoma or metastatic tumors because, to the best of our knowledge, there are no reported diagnostic features of hepatic lymphoma [13].

Pathologic findings were consistent with diffuse large B cell lymphoma in six patients, B cell lymphoma in two patients, Hodgkin’s lymphoma in two patients, and lymphocytes with interstitial fibrosis in one patient. In our case, pseudotumor is also one of the possible differential diagnoses from the result of pathology. Immunohistochemical studies of T cell and B cell subpopulations may be helpful in distinguishing inflammatory pseudotumor from lymphoma. Inflammatory pseudotumors usually contain both T cells and B cells, whereas in a lymphoma, a clonal T cell or B cell population predominates [14]. From this point, our case is consistent with B cell lymphoma. In addition, EBV is frequently associated with LPD development, and EBV positivity is observed in approximately 30–50% of all LPD cases diagnosed in patients with RA treated with MTX [8]. We considered that this case was not due to an EBV-related lymphoma secondary to immunosuppression but the direct effect of MTX since the EBV-encoded small ribonucleic acid by *in situ* hybridization of this case was negative.

Regarding treatments, to the best of our knowledge, there is no established standard method of treatment for MTX-LPD [5]. Discontinuance of MTX was done in all 11 cases. Most of the cases confirmed remission with only the discontinuance of MTX. The outcomes were: remission (*n* = 8), postoperation with no recurrence (*n* = 2), and death (*n* = 1). Recurrence occurred in one case, but chemotherapy led to remission. Operations were performed in two cases; four cases were treated with only chemotherapy. One of the patients died, and the remaining ten cases survived. When it comes to the patient who died, there are no specific characteristics compared to the other cases, but only discontinuation of MTX was done and chemotherapy was not performed as the treatment. Therefore, these findings suggest that an early diagnosis and immediate treatment, especially the discontinuance of MTX, are essential to prevent deterioration; if the lesions fail to regress, even after the discontinuance of MTX, chemotherapy should be considered.

## Conclusions

We reported a case of hepatic MTX-LPD presenting multiple hepatic lesions treated with discontinuation of MTX, and reviewed 11 previously reported cases including the present case. Physicians should discontinue MTX in patients with RA treated with MTX when elevated sIL-2R and hypovascular lesions in contrast-enhanced CT are confirmed considering the possibility of MTX-LPD.

## Data Availability

Not applicable.

## Abbreviations

CD 20: Cluster of differentiation 20
CRP: C-reactive protein
CT: Computed tomography
EBV: Epstein–Barr virus
FDG-PET: Fluorodeoxyglucose-positron emission tomography
LDH: Lactate dehydrogenase
MTX: Methotrexate
MTX-LPD: Methotrexate-associated lymphoproliferative disorders
RA: Rheumatoid arthritis
sIL-2R: Soluble interleukin-2 receptor
